# Medication stewardship by clinical pharmacists in acute ischemic stroke care: a retrospective analysis of drug-related problem reduction and cost-saving outcomes

**DOI:** 10.3389/fphar.2025.1608457

**Published:** 2025-10-07

**Authors:** Xiaoying Chen, Xiaoyan Hu, Qiang Su, Lisha Zhu, Zhiyong Tang, Chunmei Tang, Siyun Yang

**Affiliations:** ^1^ Department of Pharmacy, Nanchong Key Laboratory of Individualized Drug Therapy, Beijing Anzhen Nanchong Hospital of Capital Medical University and Nanchong Central Hospital, Nanchong, China; ^2^ Department of Pharmacy, The Second Clinical Medical College of North Sichuan Medical College, Nanchong, China

**Keywords:** clinical pharmacist, acute ischemic stroke, medication stewardship, drug-related problems, hospitalization costs

## Abstract

**Background:**

In China’s evolving Diagnosis-Intervention Packet (DIP) payment system, suboptimal medication practices in acute ischemic stroke (AIS) care exacerbate healthcare costs and antimicrobial resistance. This study evaluates the clinical and economic impacts of integrating clinical pharmacists into stroke care teams.

**Methods:**

A single-center retrospective cohort study (May–September 2024) included 439 AIS patients (clinical pharmacist care group: n = 223, standard care group n = 216). The pharmacist care group received pharmacist-led medication stewardship, including therapy optimization, adverse drug reaction (ADR) monitoring, and DIP-aligned cost management.

**Results:**

Pharmacist care significantly shortened antibiotic therapy (6.83 vs. 8.93 days, *P* = 0.019) and proton pump inhibitor (PPI) duration (7.29 vs. 9.50 days, *P* < 0.001), while reducing Ginkgolide injection use (47.53% vs. 55.56%, *P* = 0.043). Total hospitalization costs decreased by 10.4% ($1,403 ± 595.2 vs. $1,566 ± 496.0), with improved DIP settlement amount ($660.2 vs. $554.4, *P* = 0.001). Regression confirmed pharmacist intervention as an independent predictor of reduced costs and shorter stays. Medical staff reported high satisfaction with pharmacists’ roles in medication safety (84.84%) but lower recognition of cost-saving efforts (64.19%).

**Conclusion:**

Integrating clinical pharmacists into AIS care teams reduces drug-related problems (DRPs), shortens therapy duration and hospital stays, and lowers costs, supporting broader implementation in stroke management.

## 1 Introduction

Acute ischemic stroke (AIS) is a leading cause of mortality and long-term disability globally, with China bearing a disproportionately high burden due to its aging population and lifestyle shifts (Wang et al., 2017). While evidence-based therapies such as thrombolysis and antiplatelet agents form the cornerstone of AIS management, suboptimal medication practices—including excessive antibiotic use and unwarranted reliance on adjuvant therapies like Traditional Chinese Medicine (TCM) injections—persist in clinical settings (Powers et al., 2019; Huang et al., 2021b). Such practices not only escalate healthcare costs but also contribute to antimicrobial resistance (AMR) and drug related problems (DRPs), straining China’s rapidly evolving healthcare system (Formica et al., 2018; Huang et al., 2021b).

The integration of clinical pharmacists into healthcare teams has demonstrated significant success in chronic disease management, evidenced by reductions in drug-related adverse events and hospitalization costs through medication reconciliation, guideline adherence, and cost-benefit optimization (Renaudin et al., 2016; Basaraba et al., 2018). However, their role in acute neurological care, particularly in AIS, remains underexplored, despite the urgent need to address the complexities of AIS pharmacotherapy—such as time-sensitive anticoagulant/antiplatelet decision-making, post-stroke infection management, and avoidance of nonessential adjunctive therapies (Cengiz et al., 2025). This gap is compounded by China’s evolving healthcare landscape, where the 2020 introduction of the diagnosis-Intervention Packet (DIP) payment system standardized settlements based on predefined treatment bundles to curb cost inflation (Lai et al., 2022; Zhang et al., 2024; Fang and Jiang, 2025).

A significant challenge involves the extensive utilization of high-risk, low-value therapies, such as prolonged antibiotic regimens and unproven TCM injections (e.g., Ginkgolide, Danhong). These treatments constitute 15%–30% of urban hospital drug budgets, despite a lack of robust efficacy evidence (Wang et al., 2016; Li et al., 2018; Huang et al., 2021b). This misalignment underscores the necessity of pharmacist-driven stewardship programs to harmonize therapeutic practices with DIP’s value-based objectives (Phimarn et al., 2023). However, the current research predominantly emphasizes clinical endpoints (e.g., mortality, functional recovery), while overlooking comprehensive economic evaluations—such as drug-to-cost ratios, DIP balance (a metric linking diagnoses to standardized settlements), and per-case profitability—which are crucial for the scalable integration of pharmacists into acute care teams, especially in resource-constrained settings (Lai et al., 2022; Zhang et al., 2024).

To date, no studies have holistically assessed the clinical and economic impacts of pharmacist care in AIS within China’s DIP framework. This study aimed to evaluate the clinical and economic impacts of integrating clinical pharmacists into stroke care teams through a structured medication stewardship program. Specifically, we examined the program’s efficacy in optimizing high-risk medication use (antibiotics, PPIs, and TCM injections), resolving DRPs, shortening hospital stays, and reducing medical expenses.

## 2 Methods

### 2.1 Research design and ethical approval

This single-center, retrospective cohort study was conducted in the neurology department of a tertiary hospital in China between May and September 2024. This study was approved by the Ethics Committee of Beijing Anzhen Nanchong Hospital of Capital Medical University and Nanchong Central Hospital (Approval No: 2024-053). In accordance with the “Ethical Review Measures for Biomedical Research Involving Human Subjects” (2016) issued by the National Health Commission of the People’s Republic of China and institutional policies, this study is exempt from informed consent, as it permits the use of anonymized data collected retrospectively without individual consent.

### 2.2 Participants and data collection

Inclusion criteria: Patients aged ≥18 years with a disease diagnosis classified as I63.5C. Exclusion criteria: Candidates eligible for surgical interventions (e.g., mechanical thrombectomy, craniectomy); immunocompromised individuals (e.g., HIV/AIDS, undergoing chemotherapy for cancer, or receiving long-term immunosuppressive therapy); patients with incomplete medical records or non-compliant discharges. Demographic information (age, sex), comorbidities (e.g., hypertension, diabetes), and vital signs were retrieved from electronic health records. Economic outcomes included the total cost of hospitalization per case, drug cost per case, DIP settlement amount per case (the standardized reimbursement from the medical insurance bureau based on DIP grouping), and the DIP-related loss or gain per case (the difference between the actual hospitalization cost and the DIP settlement amount, indicating the hospital’s financial outcome under the DIP system). Currency conversion from Chinese Yuan (CNY) to US dollars ($) was based on an exchange rate of 7.0.

### 2.3 Intervention protocol

Patients were assigned to either the pharmacist intervention group (n = 223) or the standard care group (n = 216). In the pharmacist intervention group, trained and certified clinical pharmacists were integrated into the care team, and patients received comprehensive pharmaceutical care throughout their hospitalization. This care involved participation in care team rounds, systematic review of prescription orders (including antibiotics, PPIs, and TCM injections), continuous monitoring of drug therapy effectiveness and safety, provision of detailed information on drug efficacy and safety (e.g., ADRs), dissemination of drug-related information to physicians and nurses, and personalized medication counseling for patients. All intervention measures—such as drug discontinuation, dosage adjustment, and laboratory monitoring—and physician feedback (including whether the recommendations were adopted or rejected and the reasons for such decisions) were documented in the pharmacy intelligent monitoring system. In the standard care group, patients received routine care without pharmacist involvement or structured review of antibiotic use and adjunctive therapies.

### 2.4 Satisfaction survey

A satisfaction survey was administered to 85 medical staff members, including physicians and nurses, using a self-developed 100-point scale questionnaire. The questionnaire evaluated six domains: drug preparation and storage, patient education, ADR prevention, ward presence, cost-reduction efforts, and overall collaboration. The survey achieved a response rate of 94.11%.

### 2.5 Statistical analysis

Data were statistically analyzed using SPSS version 26 (Chicago, IL, United States). Continuous variables with normal distribution are expressed as mean ± standard deviation (SD) and compared using independent samples t-tests, while non-normally distributed variables are expressed as median (interquartile range) and compared using the Mann-Whitney U test. Categorical variables are presented as counts and percentages and compared using Pearson’s chi-squared test or continuity-corrected chi-square tests as appropriate. To control for potential confounders, multiple linear regression models were employed for continuous outcomes, including hospitalization costs, length of stay, and DIP settlement amount, with adjustments made for age, sex, insurance type, and comorbidities. A *P* value <0.05 was considered statistically significant for all analyses.

## 3 Results

### 3.1 Baseline characteristics of patients

The study comprised 439 patients (pharmacist care: n = 223; standard care: n = 216) with comparable baseline demographics and clinical characteristics (Table 1). No statistically significant differences were observed in the distribution of sex (54.7% vs. 60.6% male, *P* = 0.208), age (65.7 ± 13.8 vs. 67.2 ± 14.6 years, *P* = 0.192), anthropometric measures (BMI: 23.7 ± 4.1 vs. 23.3 ± 4.3 kg/m^2^, *P* = 0.331), comorbidities (e.g., diabetes: 38.1% vs. 38.0%, *P* = 0.984), or vital signs (e.g., systolic BP: 134.9 ± 16.8 vs. 135.3 ± 17.2 mmHg, *P* = 0.814). However, there was a significant difference in medical insurance types, with a higher proportion of inhabitants’ insurance in the standard care group (75.0% vs. 65.0%, *P* = 0.015) and staff insurance in pharmacist care group (35.0% vs. 25.0%, *P* = 0.015).

**TABLE 1:** Baseline characteristics of patients.

Parameter	Pharmacist care group	Standard care group	*P*
Sample size	223	216	—
Sex (male/female)	122 (54.7%)/101 (45.3%)	131 (60.6%)/85 (39.4%)	0.208
Age (years), mean ± SD	65.7 ± 13.8	67.2 ± 14.6	0.192
Weight (kg), mean ± SD	64.8 ± 12.9	63.5 ± 13.4	0.324
BMI (kg/m^2^), mean ± SD	23.7 ± 4.1	23.3 ± 4.3	0.331
Comorbidities			
Chronic kidney disease, n (%)	32 (14.3%)	35 (16.2%)	0.581
Chronic lung disease, n (%)	19 (8.5%)	18 (8.3%)	0.942
Cardio/cerebrovascular disease, n (%)	193 (86.5%)	186 (86.1%)	0.902
Diabetes mellitus, n (%)	85 (38.1%)	82 (38.0%)	0.984
Vital Signs			
Temperature (°C), mean ± SD	36.4 ± 0.3	36.4 ± 0.3	0.901
Pulse (bpm), mean ± SD	75.4 ± 11.9	74.6 ± 12.3	0.502
Respiratory rate (breaths/min)	18.7 ± 2.1	18.5 ± 2.0	0.418
Systolic BP (mmHg), mean ± SD	134.9 ± 16.8	135.3 ± 17.2	0.814
Diastolic BP (mmHg), mean ± SD	78.9 ± 9.8	79.2 ± 10.1	0.746
Type of medical insurance, mean ± SD			
Inhabitants	145 (65.0%)	162 (75.0%)	0.015
Staff	78 (35.0%)	54 (25.0%)	

### 3.2 DRPs, their categories and causes

DRPs categories and their associated causes are presented in Table 2. Antibiotics and PPIs were identified as having the highest DRPs rates, primarily attributed to inappropriate dosing (Antibiotics: 3.14% vs. 4.17%; PPIs: 6.73% vs. 8.33%) and excessive therapy duration (Antibiotics: 2.24% vs. 2.78%). Significant therapeutic duplication was observed for Ginkgolide injection in both the pharmacist care group (8.52%) and the standard care group (10.65%). In contrast, discrepancies in reported therapeutic duplication were noted for Danhong injection (pharmacist care: 1.35% vs. standard care 0.93%). Additionally, patients in the standard care group exhibited higher incidences of unnecessary PPI use (7.41% vs. 4.48%) and drug interactions (PPIs: 3.70% vs. 1.35%; Antibiotics: 4.17% vs. 2.69%).

**TABLE 2:** DRPs, their categories and causes.

DRPs category (n,%)	Causes of the DRPs	Pharmacist care group	Standard care group
Indication	Cause	n, %	n, %
Antibiotics	Treatment duration is longer than necessary	5/2.24%	6/2.78%
	The usage or dosage is not appropriate	7/3.14%	9/4.17%
	An antimicrobial is not indicated	3/1.35%	4/1.85%
	A therapeutic duplication exists	4/1.79%	6/2.78%
	Incompatibility or adverse interactions were observed	6/2.69%	9/4.17%
PPI	The usage or dosage is not appropriate	15/6.73%	18/8.33%
	A PPI is not indicated	10/4.48%	16/7.41%
	A therapeutic duplication exists	1/0.45%	3/1.39%
	Incompatibility or adverse interactions were present	3/1.35%	8/3.70%
Danhong injection	The selection of drugs was not appropriate	1/0.45%	3/1.39%
	A therapeutic duplication exists	3/1.35%	2/0.93%
	Incompatibility or adverse interactions were present	1/0.45%	0/0.00%
Shuxuetong injection	The selection of drugs was not appropriate	2/0.90%	2/0.93%
	A therapeutic duplication exists	1/0.45%	1/0.46%
Ginkgolide injection	The usage or dosage is not appropriate	0/0.00%	3/1.39%
	A therapeutic duplication exists	19/8.52%	23/10.65%

### 3.3 Type of pharmacist intervention

Types of pharmacist interventions and acceptance rates are presented in Table 3. The clinical pharmacist implemented a total of 173 interventions across six categories, achieving an overall acceptance rate of 75.14% (n = 130/173). These interventions encompassed six primary areas. Notably, interventions related to the evaluation of ADR achieved full acceptance (100%, n = 6/6), reflecting physicians’ high level of trust in pharmacists’ expertise for safety monitoring. The most frequent interventions pertained to adjustments in the route and dosage of administration (59 interventions, 86.44% acceptance) as well as drug selection (32 interventions, 65.63% acceptance). Conversely, interventions concerning medication indication exhibited the lowest acceptance rate (63.64%, n = 14/22), potentially indicating some reluctance among physicians to modify prescribed indications.

**TABLE 3:** Type of pharmacist intervention.

Intervention type	Number of interventions (N)	Number of interventions received (n)	Intervention acceptance rate (%)
Route and dosage of administration	59	51	86.44%
Duration of medication treatment	28	20	71.43%
Evaluation of ADR	6	6	100.00%
Indication for medication	22	14	63.64%
Drug selection	32	21	65.63%
Inappropriate combination of drugs	26	18	69.23%
Total	173	130	75.14%

### 3.4 Categories of clinical pharmacist care and their impact on health outcomes

As shown in Table 4, the interventions conducted by clinical pharmacists significantly decreased the duration of antibiotic therapy (6.83 ± 3.23 vs. 8.93 ± 4.36 days, *P* = 0.019) and PPI therapy (7.29 ± 3.91 vs. 9.50 ± 4.00 days, *P* < 0.001). Additionally, these interventions led to a reduced utilization of Ginkgolide injection compared to the standard care group (47.53% vs. 55.56%, *P* = 0.043). Although no significant differences were observed in the utilization rates of antibiotics and PPIs between the two groups (*P* > 0.05), clinical pharmacist care group exhibited shorter hospital stays (9.54 vs. 10.5 days, *P* = 0.028) and a shorter duration of Shuxuetong injection treatment (6.30 ± 3.92 vs. 7.10 ± 4.33 days, *P* = 0.031). Furthermore, the incidence of adverse drug reactions demonstrated a non-significant downward trend (8.07% vs. 11.57%, *P* = 0.201), while the proportion of drug costs remained comparable between the groups (24.16% vs. 25.86%, *P* = 0.412).

**TABLE 4:** Comparison of health outcomes.

Types of indicators		Pharmacist care group (n = 223)	Standard care group (n = 216)	*P*
Primary outcomes				
Antibiotics uses	Utilization rate of antibiotics (n/%)	32/14.35	40/18.52	0.236
	Course of antibiotic therapy (days)	6.83 ± 3.23	8.93 ± 4.36	0.019
	Microbial submission rate (n/%)	50/22.42	50/23.15	0.892
PPI uses	PPI utilization rate (n/%)	63/28.25	63/29.17	0.85
	PPI treatment course (days)	7.29 ± 3.91	9.50 ± 4.00	0.001
TCM injection use	Utilization rate of Danhong injection (n/%)	87/39.01	88/40.74	0.722
	Danhong injection treatment course (days)	7.22 ± 4.07	7.10 ± 4.29	0.826
	Utilization rate of Shuxuetong injection (n/%)	84/37.67	91/42.13	0.339
	Shuxuetong injection treatment course (days)	6.30 ± 3.92	7.10 ± 4.33	0.031
	Utilization rate of Ginkgolide injection (n/%)	106/47.53	120/55.56	0.043
	Ginkgolide injection treatment course (days)	6.30 ± 3.92	7.85 ± 9.59	0.031
Adverse drug reactions	Incidence of adverse drug reactions (n/%)	18/8.07	25/11.57	0.201
Secondary outcomes				
Length of stay (median, days)		9.54	10.5	0.028
Proportion of drugs (%)		24.16	25.86	0.412
Number of national essential medicines		8.11 ± 3.38	7.89 ± 3.05	0.085

### 3.5 Effect of clinical pharmacist intervention on economic indicator in patients with AIS

Economic outcomes are reported in Table 5. The incorporation of clinical pharmacists into the management of ischemic stroke patients resulted in a significant reduction in healthcare expenditures. Specifically, the total hospitalization cost per case was 10.4% lower in clinical pharmacist care group compared to the standard care group ($1,403 ± 595.2 vs. $1,566 ± 496.0, *P* = 0.001), and the drug cost per case decreased by 13.5% ($360.9 ± 232.1 vs. $417.3 ± 202.4, *P* = 0.005). Furthermore, clinical pharmacist care group exhibited higher DIP settlement amounts ($660.2 ± 308.3 vs. $554.4 ± 360.3, *P* = 0.001), along with a non-significant downward trend in DIP losses per case ($104.1 ± 495.0 vs. $152.2 ± 455.7, *P* = 0.331). These results underscore the critical role of clinical pharmacists in optimizing treatment costs and enhancing settlement amount.

**TABLE 5:** Economic indicator comparison (the unit is $).

Types of indicators	Pharmacist care group ($)	Standard care group ($)	*P*
Total cost of hospitalization per case	1,403 ± 595.2	1,566 ± 496.0	0.001
DIP settlement amount per case	660.2 ± 308.3	554.4 ± 360.3	0.001
Amount loss of DIP per case	104.1 ± 495.0	152.2 ± 455.7	0.331
Drug cost per case	360.9 ± 232.1	417.3 ± 202.4	0.005

### 3.6 Independent association of pharmacist intervention with outcomes

To control for potential confounding variables and identify independent predictors, multiple linear regression analyses were conducted, adjusting for these factors (Table 6). Following adjustment, clinical pharmacist intervention remained a significant independent predictor of reduced total hospitalization costs (B = −175.77, 95% CI: -281.6 to −69.9, *P* = 0.001) and shorter length of stay (B = −0.91, 95% CI: -1.61 to −0.22, *P* = 0.010). Conversely, the intervention was independently associated with an increase in DIP settlement amount (B = 116.39, 95% CI: 50.8 to 182.0, *P* < 0.001). No other covariates showed statistically significant associations with the outcomes in the adjusted models.

**TABLE 6:** Results of multiple linear regression analyses assessing the impact of clinical pharmacist intervention.

Outcome variable	Predictor	Unstandardized B	Standardized β	P value	95% CI for B
Total Cost ($)	Group	−175.77 (53.86)	−0.159	0.001	[−281.6, −69.9]
	Age	−1.48 (2.47)	−0.029	0.549	[−6.34, 3.38]
	Sex	30.04 (54.68)	0.027	0.583	[−77.5, 137.6]
	Comorbidity	−96.05 (64.24)	−0.072	0.136	[−222.5, 30.4]
	Insurance Type	−41.62 (59.15)	−0.034	0.482	[−158.0, 74.8]
Length of Stay (Days)	Group	−0.91 (0.36)	−0.126	0.01	[−1.61, −0.22]
	Age	−0.01 (0.02)	−0.022	0.658	[−0.04, 0.02]
	Sex	−0.32 (0.36)	−0.043	0.383	[−1.02, 0.39]
	Comorbidity	0.11 (0.42)	0.012	0.805	[−0.73, 0.94]
	Insurance Type	−0.31 (0.39)	−0.039	0.431	[−1.08, 0.46]
DIP Amount ($)	Group	116.39 (33.38)	0.169	<0.001	[50.8, 182.0]
	Age	−0.28 (1.53)	−0.009	0.854	[−3.29, 2.73]
	Sex	−12.75 (33.89)	−0.018	0.707	[−79.5, 54.0]
	Comorbidity	73.90 (39.81)	0.09	0.064	[−4.4, 152.2]
	Insurance Type	−32.05 (36.66)	−0.043	0.382	[−104.2, 40.1]

### 3.7 The satisfaction of medical staff to the service provided by clinical pharmacists

**FIGURE 1:**
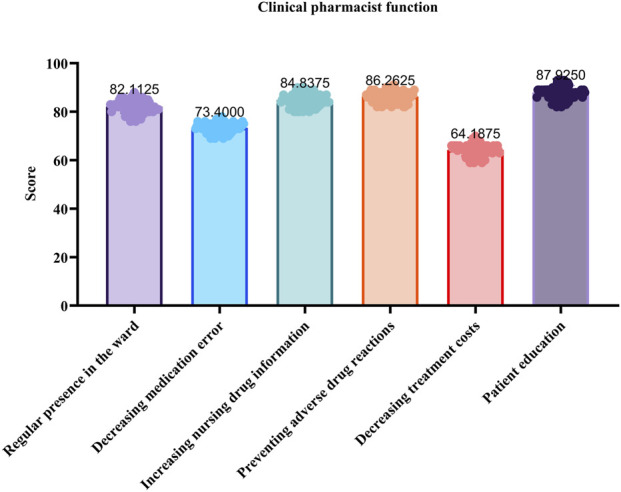
Medical staff satisfaction with the services provided by clinical pharmacists.

Figure 1 illustrates the level of satisfaction among neurology medical staff regarding the services provided by clinical pharmacists. Healthcare providers expressed high levels of satisfaction with the functions performed by clinical pharmacists, particularly in areas such as drug preparation, storage, and administration (87.93%) as well as patient education (86.26%). These findings highlight the critical role of pharmacists in ensuring medication safety and promoting patient-centered care. Additionally, core clinical contributions, including the prevention of adverse drug reactions (84.84%) and consistent presence in the ward (82.11%), were highly appreciated. However, satisfaction with the reduction of treatment costs was relatively lower (64.19%), suggesting potential gaps in cost-reduction strategies or interdisciplinary collaboration that warrant further exploration.

## 4 Discussion

Within China’s evolving DIP payment system—implemented in 2020 to standardize reimbursements and control healthcare costs—suboptimal medication practices in acute ischemic stroke AIS care, such as antibiotic overuse and unnecessary TCM injections, remain prevalent (Lai et al., 2022; Huang et al., 2021b). The study identified antibiotics as a major contributor to DRPs, with inappropriate dosing (3.14% vs. 4.17%) and prolonged therapy duration (2.24% vs. 2.78%) being the primary issues. These findings align with global antimicrobial stewardship literature, where 30%–50% of antibiotic prescriptions are deemed inappropriate due to dosing errors, unnecessary duration, or lack of indication (Tamma et al., 2017). Pharmacist-led interventions in this study reduced antibiotic treatment duration by 2.1 days (6.83 vs. 8.93 days, *P* = 0.019), corroborating the Infectious Diseases Society of America guidelines, which emphasize pharmacist roles in optimizing antibiotic use to curb resistance (Tamma et al., 2021; Dighriri et al., 2023). A meta-analysis by Davey et al. further supports this, showing that pharmacist interventions shorten antibiotic courses by 1.5–3 days and reduce *Clostridium difficile* infection rates by 18% (Davey et al., 2017). Excessive PPI use, particularly in the standard care group (7.41% vs. 4.48%, *P* < 0.05), mirrors global trends where 70% of PPI prescriptions lack appropriate indications (Scarpignato et al., 2016). Pharmacist interventions reduced PPI therapy duration by 2.21 days (7.29 vs. 9.50 days, *P* < 0.001), consistent with deprescribing trials by Reeve et al., which reported a 40% reduction in PPI use among elderly patients (Reeve et al., 2017). The economic burden of PPI overuse is substantial, costing 1.2–2.4 billion annually in the U.S. alone due to adverse events like fractures and renal dysfunction (Forgacs and Loganayagam, 2008). In this study, pharmacists lowered drug costs by 13.5%, aligning with Perez et al., who estimated $1,200 annual savings per patient through pharmacist-driven deprescribing (Mi et al., 2020).

Therapeutic duplication of Ginkgolide (8.52% vs. 10.65%) and Danhong injections (1.35% vs. 0.93%) underscores the need for evidence-based TCM prescribing. While Ginkgolide is widely used in Asia for ischemic stroke, its efficacy remains contentious. A Cochrane review by Wu et al. found insufficient evidence to support its superiority over placebo in improving neurological outcomes (Ji et al., 2020). While Danhong injection has shown modest benefits in microcirculation improvement (Shi et al., 2025), its use is often conflated with conventional therapies. It has been reported in the literature that 22% of TCM injections were prescribed without clear clinical indications, highlighting the critical need for comprehensive pharmacovigilance systems and strict adherence to clinical guidelines (Feng et al., 2019; Huang et al., 2021a).

Pharmacist interventions significantly reduced hospital stays by 0.96 days (9.54 vs. 10.5 days, *P* = 0.028), translating to an estimated cost saving of 1,200 perpatientbasedonper−diemhospitalizationcosts. This improved DIP settlement amounts ($660.2 ± 308.3 vs. $554.4 ± 360.3, *P* = 0.001), who reported cost savings via pharmacist involvement (Feldman et al., 2025)—and reimbursement efficiency driven by pharmacist adherence to standardized protocols under DIP system, a strategy shown by Yip et al. (2019) to increase reimbursements by 15%. The results of the multiple linear regression analysis indicated that pharmacist intervention was a significant independent predictor of both lower total hospitalization costs (B = −175.77, 95% CI: -281.6 to −69.9, *P* = 0.001) and shorter length of stay (B = −0.91, 95% CI: -1.61 to −0.22, *P* = 0.010), even after adjusting for potential confounding variables. However, the non-significant reduction in adverse drug reactions (8.07% vs. 11.57%, *P* = 0.201) underscores the need for integrating real-time ADR monitoring tools to harmonize cost containment with patient safety priorities (Buttigieg et al., 2020).

High satisfaction rates with pharmacist roles in drug preparation (87.93%) and patient education (86.26%) align with global trends, as exemplified by Zillich et al. (2004), who reported that 82% of physicians valued pharmacists’ input on dose adjustments—a finding consistent with the 86.4% acceptance rate for administration/dosage interventions observed in this study. However, systemic barriers persist, particularly in cost-reduction strategies (64.19% satisfaction) and indication-related interventions (63.6% acceptance). These challenges stem from hierarchical resistance, where physicians may perceive indication modifications as encroachments on clinical autonomy, as documented in Japanese healthcare settings (Tasaka et al., 2016), and communication gaps arising from siloed workflows that hinder multidisciplinary consensus on cost-saving measures (Bondesson et al., 2013). To address these barriers, models such as interprofessional co-management, shared cost-saving performance indicators, and interdisciplinary training programs have been introduced to enhance mutual understanding and cooperation among healthcare professionals. Expanding the scope of practice for pharmacists and further integrating them into healthcare teams can significantly reduce medication errors, thereby improving patient outcomes and the quality of healthcare delivery (Gillani et al., 2021). Another study found that embedding pharmacists within hospital wards improved team satisfaction by 22% in Australian contexts (Pouranayatihosseinabad et al., 2018). These strategies highlight the importance of structural collaboration in bridging disciplinary divides and enhancing the uptake of clinical interventions.

This study has limitations, including its single-center design, potential confounding from insurance-type disparities, and lack of long-term clinical outcome assessments (e.g., mortality, readmission rates). The imbalance in medical insurance types between groups (*P* = 0.015) may confound economic outcomes, as different insurance types entail varying reimbursement rates and patient cost-sharing structures. Although we adjusted for insurance type in regression analyses, residual confounding may remain. Future research should prioritize multicenter randomized controlled trials to validate generalizability, integrate societal cost-effectiveness analyses (e.g., productivity losses), and AI-enabled DRPs detection to enhance scalability and precision. These steps will strengthen the evidence base for pharmacist-led interventions in evolving healthcare systems.

## 5 Conclusion

The integrating clinical pharmacists into multidisciplinary care for acute ischemic stroke patients enhances drug therapy outcomes and resource efficiency, evidenced by reduced hospitalization costs, improved reimbursement under China’s DIP system, and shortened antibiotic/PPI durations. Their specialized knowledge in resolving DRPs and aligning therapeutic strategies with value-based care paradigms establishes clinical pharmacists as critical participants within contemporary healthcare systems.

## Data Availability

The original contributions presented in the study are included in the article/supplementary material, further inquiries can be directed to the corresponding author.
